# Construction Notes

**Published:** 1907-04-06

**Authors:** 


### CONSTRUCTION NOTES.

### BUCHANAN HOSPITAL, ST. LEONARDS.

Very heavy expenses have been incurred in order to put the drains and sanitary arrangements of the hospital in order. Additional lavatory accommodation has been provided in all the wards, and the sculleries and ward kitchens have been altered and enlarged.

### CARDIFF INFIRMARY.

This institution, which recently appealed for funds to remodel its out-patient department, has now actively embarked upon its new building scheme, the larger portion of the building fund having been subscribed. From the annual report just to hand it seems that the scheme which the board suggests will provide sixty-four additional ordinary beds, and six beds for isolation purposes. The necessity for isolation beds has been seriously felt for some years, and quite recently an outbreak of measles occurring in the institution was dealt with under great difficulty. The medical staff has in consequence expressed the opinion that in view of the present size of the infirmary the erection of an isolation block has become an urgent necessity. There will be a central lift for patients, which will greatly facilitate the work of the hospital and the careful removal of the patients from the operating theatre to the wards, so essential after an operation. The erection of a corridor connecting the wards on the first floor and the lifts is also provided for. Provision is made for a new gynæcological operating-room—the existing theatre can only be used one day a week for these cases—and for a clinical laboratory, small museum, and new post-mortem room, with modern adjuncts. It is proposed to build these on the eastern boundary of the site, and the rooms which are now used in the main building for this purpose can then be utilised for the purpose for which they were originally intended. The approximate cost of the buildings, including lighting, heating, and furniture, will be as follows: Pavilion for sixty-four beds, plus six beds for isolation purposes, including the nurses' and servants' bedrooms, £22,500; new gynæcological operating-room, £2,500; corridor and lifts to connect the wards on the first floor, £2,000; clinical laboratory, museum, and new post-mortem room, £3,000—total, £30,000.

### NORTH DEVON INFIRMARY.

The improvement scheme has been fully considered, and the new buildings are now practically completed. The additions consist primarily of two new sanitary towers with an isolation ward with separate offices, and disconnected from the main building. In addition, many of the wards have been enlarged and rearranged, to ensure the maximum amount of comfort for the inmates with the minimum working expense of staff. The lavatories are entirely disconnected from the wards by cross-ventilated lobbies, and, in fact, effective ventilation has been one of the chief studies, combined with cleanliness. To ensure the latter, new floors have been put in with "coved" skirting. The lack of proper means of escape in case of fire has been remedied by the provision of two fire-escape staircases outside the building. At considerable expense the drainage system has been remodelled, and new ideas introduced. Internally the heating system has been extended and improved, a new hot-water system fitted up, and four new baths put in for the exclusive use of the patients. On many of the ceilings a fire-resisting material has been used, whilst generally every modern idea has been carried into effect in regard to fittings and appliances. Another most important improvement has been effected in the water service. The whole work was commenced in June 1906, and each section as completed was handed over to the hospital staff for use.

